# Geosocial Media’s Early Warning Capabilities Across US County-Level Political Clusters: Observational Study

**DOI:** 10.2196/58539

**Published:** 2025-01-30

**Authors:** Dorian Arifi, Bernd Resch, Mauricio Santillana, Weihe Wendy Guan, Steffen Knoblauch, Sven Lautenbach, Thomas Jaenisch, Ivonne Morales, Clemens Havas

**Affiliations:** 1 Department of Geoinformatics University of Salzburg Salzburg Austria; 2 Interdisciplinary Transformation University Austria Linz Austria; 3 Center for Geographic Analysis (CGA) Harvard University Cambridge, MA United States; 4 Machine Intelligence Group for the Betterment of Health and the Environment Northeastern University Boston, MA United States; 5 Department of Epidemiology Harvard TH Chan School of Public Health Boston, MA United States; 6 GIScience Research Group Heidelberg University Heidelberg Germany; 7 Interdisciplinary Centre of Scientific Computing (IWR) Heidelberg University Heidelberg Germany; 8 HeiGIT (Heidelberg Institute for Geoinformation Technology) GbmH Heidelberg Germany; 9 Center for Global Health Colorado School of Public Health Aurora, CO United States; 10 Heidelberg Institute of Global Health Heidelberg University Hospital Heidelberg Germany; 11 Department of Infectious Disease and Tropical Medicine Heidelberg University Hospital Heidelberg Germany; 12 Salzburg University of Applied Sciences Puch/Salzburg Austria

**Keywords:** spatiotemporal epidemiology, geo-social media data, digital disease surveillance, political polarization, epidemiological early warning, digital early warning

## Abstract

**Background:**

The novel coronavirus disease (COVID-19) sparked significant health concerns worldwide, prompting policy makers and health care experts to implement nonpharmaceutical public health interventions, such as stay-at-home orders and mask mandates, to slow the spread of the virus. While these interventions proved essential in controlling transmission, they also caused substantial economic and societal costs and should therefore be used strategically, particularly when disease activity is on the rise. In this context, geosocial media posts (posts with an explicit georeference) have been shown to provide a promising tool for anticipating moments of potential health care crises. However, previous studies on the early warning capabilities of geosocial media data have largely been constrained by coarse spatial resolutions or short temporal scopes, with limited understanding of how local political beliefs may influence these capabilities.

**Objective:**

This study aimed to assess how the epidemiological early warning capabilities of geosocial media posts for COVID-19 vary over time and across US counties with differing political beliefs.

**Methods:**

We classified US counties into 3 political clusters, democrat, republican, and swing counties, based on voting data from the last 6 federal election cycles. In these clusters, we analyzed the early warning capabilities of geosocial media posts across 6 consecutive COVID-19 waves (February 2020-April 2022). We specifically examined the temporal lag between geosocial media signals and surges in COVID-19 cases, measuring both the number of days by which the geosocial media signals preceded the surges in COVID-19 cases (temporal lag) and the correlation between their respective time series.

**Results:**

The early warning capabilities of geosocial media data differed across political clusters and COVID-19 waves. On average, geosocial media posts preceded COVID-19 cases by 21 days in republican counties compared with 14.6 days in democrat counties and 24.2 days in swing counties. In general, geosocial media posts were preceding COVID-19 cases in 5 out of 6 waves across all political clusters. However, we observed a decrease over time in the number of days that posts preceded COVID-19 cases, particularly in democrat and republican counties. Furthermore, a decline in signal strength and the impact of trending topics presented challenges for the reliability of the early warning signals.

**Conclusions:**

This study provides valuable insights into the strengths and limitations of geosocial media data as an epidemiological early warning tool, particularly highlighting how they can change across county-level political clusters. Thus, these findings indicate that future geosocial media based epidemiological early warning systems might benefit from accounting for political beliefs. In addition, the impact of declining geosocial media signal strength over time and the role of trending topics for signal reliability in early warning systems need to be assessed in future research.

## Introduction

On March 12, 2020, the World Health Organization (WHO) declared the novel coronavirus disease COVID-19 a pandemic [1]. Its high infectiousness and severity posed a great threat to large populations worldwide, ultimately causing an estimated 15.9 million pandemic-related deaths [2], challenging health care professionals, hospitals, and authorities alike. Thus, decision makers around the world sought to unravel and predict the spreading dynamics of this novel coronavirus. Consequently, researchers explored various ways of adjusting and improving existing epidemiological early warning systems, with complementary internet-based data sources being one such method to better monitor and anticipate how this new disease would affect different geographies around the world [3-5].

Multiple studies have already emphasized the role of geosocial media data in improving early warning of epidemiological phenomena. For instance, geosocial media data were used to improve real-time reporting on diseases like Zika and Ebola [6] or to enhance the prediction of dengue fever [7]. Accordingly, various recent examples further emphasize the ability of geosocial media data for real-time surveillance and early warning in the context of COVID-19 [8,9]. In this regard, Kogan et al [10] observed that in the beginning of the pandemic, increases in geosocial media activity, among other digital data sources, preceded surges in COVID-19 cases by 2 to 3 weeks on state level. Similarly, Zhang et al [11] used geosocial media posts in a linear regression model to predict COVID-19 signals on state-level. Yet, an increasing trend in epidemiological analysis focuses on ever finer spatial scales in the hopes of gaining a more distinct understanding of infection patterns. In this regard, Stolerman et al [12] investigated the value of X posts (formerly known as Twitter) for COVID-19 early warning on a representative subset of US counties. However, the authors only investigated a comparably small sample of counties (n=97), raising questions with respect to the generalizability of the presented results. Thus, in this study, we extended their investigation on the early warning capabilities of geosocial media data to all US counties.

Furthermore, geosocial media data garnered notable attention across various fields to answer research questions related to mental health or public attitudes, during the COVID-19 pandemic [13]. For instance, researchers investigated how language in Reddit posts reflected real-world pandemic-driven events like lockdowns, revealing significant psychological shifts among users which coincided with tendencies toward decreased analytical thinking [14]. Similarly, Swain et al [15] developed a machine learning model leveraging geosocial media data to predict disruptions in mental well-being caused by the COVID-19 pandemic. Beyond that, researchers explored geosocial media users’ attitudes and concerns toward COVID-19 vaccines for the United States and the United Kingdom [16]. They observed that geosocial media derived results correlated broadly with nationwide surveys. In essence, the previous results suggest that geosocial media exchange during the COVID-19 pandemic was likely influenced by real-world public attitudes and even users’ mental health. Similarly, a variety of studies indicate that the language used and the topics of interest of geosocial media users vary based on political beliefs [17-19]. This further supports our underlying assumption that differences in political beliefs are likely to be reflected in geosocial media behavior, which could, in turn, correspond to differences in geosocial media’s early warning capabilities for COVID-19 cases.

However, even before the surge of the COVID-19 pandemic, researchers observed the emergence of echo chambers when analyzing pro and antivaccination attitudes on Facebook (Meta), which in their opinion might have caused further polarization [20]. In this regard, Howard et al [21] found that X was particularly prone to misinformation and polarizing content compared with professionally produced news during the 2016 presidential election. They even found more misinformation being prevalent in swing states. Such spread of misinformation and emerging political polarization on geosocial media should be of further concern for health experts and policy makers. In particular, since many researchers illustrated that diverging political beliefs can not only influence exchange on geosocial media [17-19], but also real-world individual behavior such as vaccine up-take [22] or the usage of nonpharmaceutical interventions such as mask wearing [23]. This is in line with previous findings [24], which highlight significant variation between individuals with different political beliefs with respect to self-estimated COVID-19 risks, self-reported adherence to COVID-19 health care measures, and expectations on the future course of the pandemic. In addition, researchers observed that US counties that voted in favor of the republican presidential candidate in the 2016 election, experienced up to 3 times higher mortality due to COVID-19 during the winter of 2020 [25].

Hence, in essence it can be assumed that individuals may respond differently on geosocial media to a swiftly politicized epidemic event like the COVID-19 pandemic [26], corresponding to their political beliefs. Evidence further suggests that differences in political beliefs do not only influence online and offline behavior, but they might indeed coincide with higher COVID-19 cases and death rates [25,27,28]. In summary, these results highlight the need to understand and adjust geosocial media based early warning systems with respect to political beliefs. Thus, within the scope of this paper, we seek to answer the following 2 research questions with a particular focus on geosocial media posts:

1. How do the early warning capabilities of geosocial media data change across consecutive epidemiological waves of COVID-19 cases?
2. What differences across US county-level political clusters can be observed with respect to geosocial media’s early warning capabilities for COVID-19 cases?

To explore the early warning capabilities of geosocial media data, we determined the correlation between geosocial media posts and COVID-19 cases and the number of days by which signals in geosocial media data preceded actual COVID-19 cases (temporal lag). Furthermore, we specifically examined the temporal lag and the correlation in the context of political clusters based on US county voting data and over the course of 6 consecutive waves of COVID-19 cases.

## Methods

### Data Collection

We used 2 main data sources in this study. First, we gathered official data on confirmed COVID-19 cases in the United States and we obtained geolocated posts (Tweets) from the geosocial media network X. The time frame for which we collected our data ranges from February 28, 2020, the beginning of the pandemic in the United States, to April 27, 2022, which denotes the end of the first major Omicron wave that began in November 2021 [29]. This time frame covers the main COVID-19 waves, time periods before and after the availability of vaccines, and was selected based on retrospective knowledge on the course of the pandemic. The contiguous United States was chosen as our study area. Furthermore, to gain a more refined understanding of the underlying spatial patterns, we decided to use US counties as our finest spatial analysis resolution, on which we identified politically similar clusters, advancing previous research that was mostly performed on national or state levels.

### COVID-19 Case Data

We downloaded officially confirmed COVID-19 cases for the United States in csv format from the not-for-profit public data aggregator USAFacts [30]. The COVID-19 cases csv file contained daily cumulated COVID-19 cases, which we transformed into daily incidence data. In addition, we applied a 14-day moving average to account for possible reporting delays and differing update cycles across states.

### Geosocial Media Data

Furthermore, we collected geolocated posts from the geosocial media network X through their official application programming interfaces (APIs) during our investigation time frame [10,12], when academic access for researchers was still available. In particular, we used the Twitter REST and Streaming API access points to gather about 727 million geosocial media posts. The REST API allowed us to retrieve posts from the previous 7 days, with a limit of 450 requests per 15-minute window. In contrast, the Streaming API provided a continuous, real-time stream of posts. For both API endpoints we applied filters to capture only posts containing a geolocation. Thus, each collected geosocial media post includes a geolocation, which can either be the Global Navigation Satellite System position of the device through which the post was shared, or a user-defined location. Furthermore, locations can consist of polygons (eg, city, state level polygons) or point locations. We excluded geosocial media posts with polygon or point geometries that were not located within the county-level geometries, which left us with 242 million posts.

Next, to obtain geosocial media posts that are relevant to the analysis of COVID-19, we performed keyword filtering on the remaining 242 million posts located within county geometries. Therefore, we defined keywords based on the knowledge of geosocial media and health experts, with the goal to properly capture geosocial media trends relevant to the COVID-19 pandemic (Textbox 1). For some keywords only their word stem was used to allow for different variations of the word to be detected.

**Keywords used for relevant post extraction.**

COVID-19 keywords:

covid, corona, sarscov, sars-cov, sars, epidemic, pandemic, influenza, virus, viral, infect, spread, 2019-ncov, Delta variant, Omicron, H1N1, H3N2, Wuhan, sickness, transmission, contagio, Illness, outbreak, super spread, incubation, quarantine, lockdown, vaccin, fever, cough, headache, fatigue, body aches, loss of taste, loss of smell, no smell, no taste, respirator, face mask, masks.

After the keyword extraction, the posts were aggregated on US county-level and a 14-day moving average was applied. Finally, to cope with differing amounts of geosocial media posts over time and space, we normalized the amount of relevant filtered geosocial media posts over the amount of all geosocial media posts on county level. In the remainder of this study, we solely used this ratio, that is, the proportion of relevant posts over all posts per county. This allows us to account for spatially clustered population and post density. In total, the semantic filtering procedure left us with 3.3 million relevant posts.

### Political Clusters

**Figure 1:**
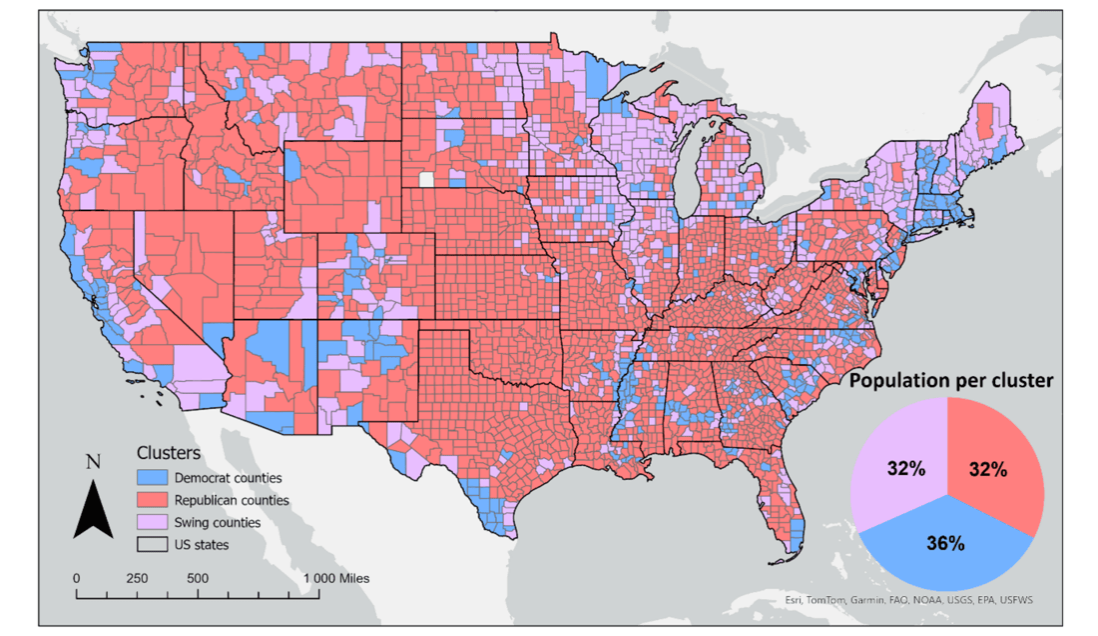
Geospatial distribution of political belief clusters on county level based on the last 6 election cycles.

To examine the differences between the various political beliefs, we based our analysis on voting data from the last 6 US presidential elections. The voting data were obtained from the Harvard Dataverse [31]. We classified US counties into 3 different clusters depending on their historical vote share for either the republican or the democrat party. In the political sciences literature, swing states are traditionally defined through a variety of quantitative and qualitative indicators. However, most of these definitions such as the bellwether status of a state [32], or it being perceived as a battleground [32], are not directly transferable to county-level analysis. Thus, we decided to base the classification into republican, democrat, or swing county clusters, on the so-called flippability of a county [32]. We chose to assess the flippability of a county on its last 6 federal election cycles. Concretely, we classified a county as belonging to a specific party, if said party had won at least 5 consecutive elections in the last 6 elections cycles. All other counties were considered as flipping between political parties and thus classified as swing counties. This division yielded political clusters, each of which representing approximately one third of the US population (Figure 1). 

### Defining COVID-19 Waves

We split the COVID-19 cases time series into smaller time frames, to capture individual epidemiological waves. However, there exist multiple approaches to define epidemic waves ranging from statistical methods using, for instance, exponential growth [10,33] or the effective reproduction number R [12,34]. In contrast, other authors tried to identify statistics and guiding principles on the duration of COVID-19 waves based on empirical data [35]. Nevertheless, all these approaches are based on strong assumptions and subjective definitions on what thresholds characterize an epidemic wave. Thus, similarly to [35], we based our definition of COVID-19 waves on a rule-based approach using the local minima on a 21-day moving average of the COVID-19 cases, which was informed through retrospective knowledge on the course of the pandemic.

**Figure 2:**
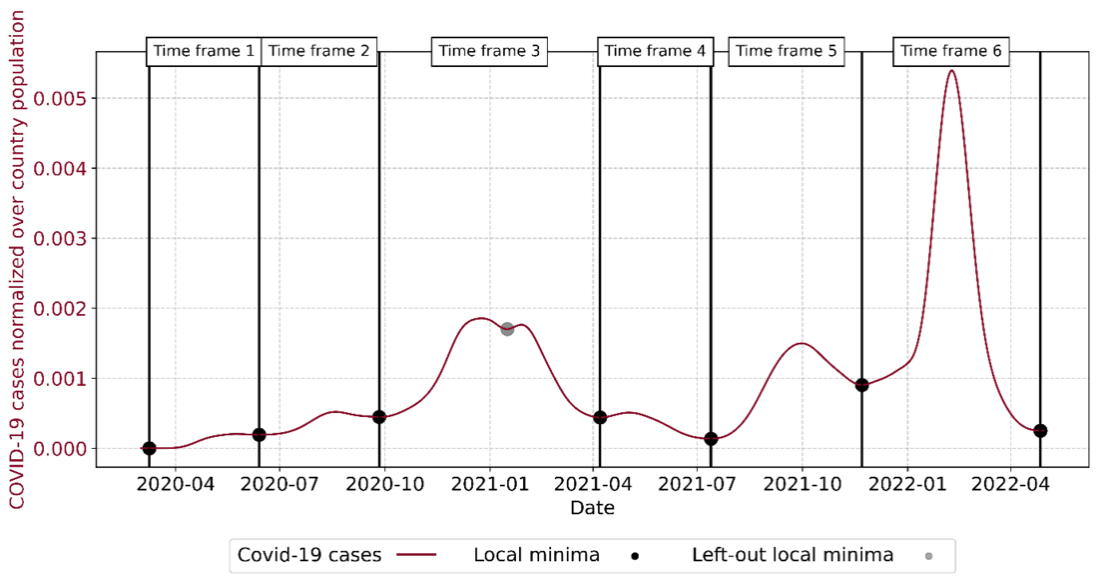
COVID-19 case waves for the entire US primarily defined through local minima.

We defined these time frames based on COVID-19 cases for the entire United States, rather than defining them individually for each political cluster. Furthermore, our procedure yielded 7 different time frames (Figure 2). Nonetheless, these 7 time frames did not accurately reflect all epidemic waves. In particular, the wave ranging roughly from October 2020 to April 2021, was split into 2. As a result, we decided to combine the original time frames 3 and 4 into 1 epidemic wave, which left us with 6 epidemic waves in total. This decision enabled us to capture the epidemic waves more accurately (Figure 2).

### Early Warning Capabilities

Finally, we quantified the early warning capabilities separately for each of the epidemic waves. We defined early warning capabilities twofold: (1) as the Pearson correlation between the time series of COVID-19 related geosocial media posts and COVID-19 cases, and (2) the number of days by which geosocial media posts preceded COVID-19 cases. However, the more important measure for early warning is the correlation between the 2 time series. Put differently, this means that if the temporal lag is high, however a correlation close to zero is present, it is obviously not reasonable to attribute any early warning capabilities to geosocial media data.

Furthermore, to identify the maximal correlation and the corresponding temporal lag, we shifted the geosocial media posts time series between 7 and 42 days into the future to determine the highest possible early warning capabilities. This procedure is repeated for each individual political cluster and epidemic wave, respectively. The decision to investigate a temporal lag between 7 and 42 days into the future was based on previous results [12], in which an early warning model, using, among others, geosocial media data, was able to predict COVID-19 cases between 1 and 6 weeks in advance.

### Ethical Considerations

The study was carried out in accordance with the Declaration of Helsinki and with the ethical regulations in place at the Paris Lodron University of Salzburg, and complies with the General Data Protection Regulation legislation of the European Union. We only used publicly available data, which were collected in accordance with the terms of service of the respective geosocial media platform X at the time of data collection. Furthermore, no identifiable information was revealed in this study. Specifically, the user-provided geographic locations and semantic content were spatially aggregated to ensure user privacy and anonymity. Thus, we did not need to seek ethical approval from our institution for this study.

## Results

### Democrat Counties

**Figure 3:**
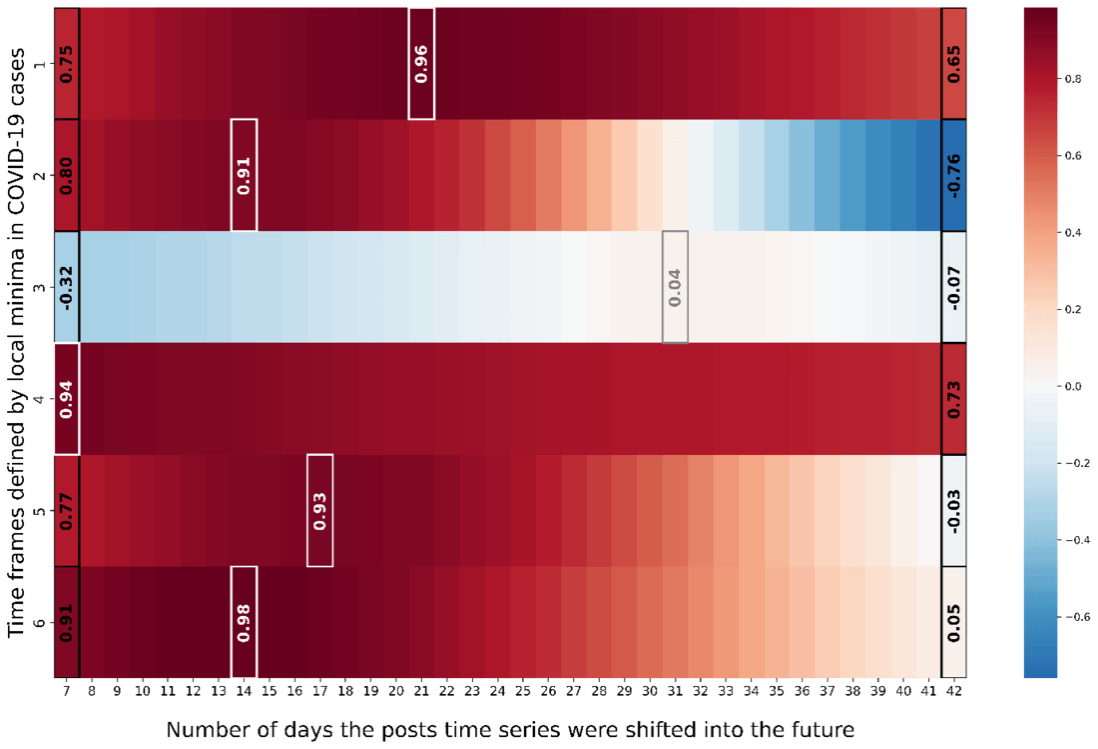
Depicting the Pearson correlation between COVID-19 cases and geosocial media post time series for each epidemiological wave when stepwise shifting the geosocial media post time series into the future for democrat counties.

**Figure 4:**
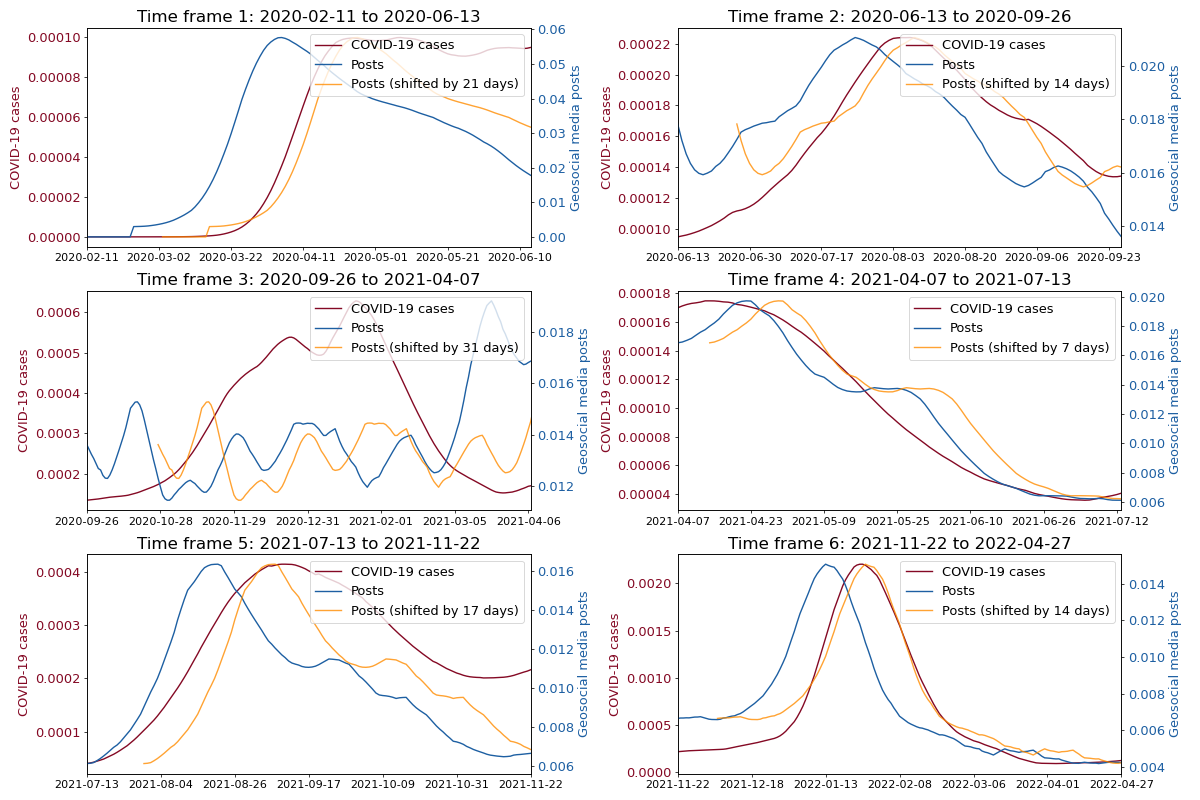
Depicting COVID-19 cases and the geosocial media posts time series and the shifted geosocial media posts time series across individual epidemic waves for democrat counties.

Figure 3 depicts the Pearson correlation for different temporal lags between the time series of COVID-19 cases and geosocial media posts in democrat counties. In particular, the y-axis represents the individual waves of COVID-19 cases as introduced in Figure 2, while the x-axis denotes the number of days the posts time series was shifted into the future. The coloring of individual windows reflects the Pearson correlation between COVID-19 cases and the shifted posts time series. Furthermore, Figure 4 illustrates the corresponding COVID-19 cases, the post time series and the post time series shifted by the correlation maximizing temporal lag for each individual epidemic wave.

The results for democrat counties in Figure 3 indicate the highest Pearson correlations between posts and COVID-19 cases time series in 5 out of 6 epidemic waves, for a shift of 7 to 21 days (time frames 1, 2 and 4-6). For the same 5-time frames, the Pearson correlations ranged between 0.91 to 0.98. Furthermore, Figures 3 and 4 suggest that only for time frames 1, 2 and 4-6, geosocial media data exhibited actual early warning capabilities. For these time frames, signals in COVID-19 cases were clearly preceded by signals in X data, while for time frame 3 no clear early warning signal in geosocial media data was apparent. Nevertheless, in the beginning of the pandemic (time frames 1 and 2) geosocial media posts showcased a clear increase up to 21 (time frame 1) and 14 days (time frame 2) ahead increases in COVID-19 infections, with Pearson correlations of 0.96 and 0.91. In addition, the COVID-19 wave from mid of July 2021 to the end of November 2021 (time frame 5) was reflected in geosocial media posts up to 17 days earlier than an increase in COVID-19 cases, with a Pearson correlation of 0.93. Also, the Omicron wave (time frame 6) starting in mid of November 2021 [29] was accurately reflected 14 days in advance in the geosocial media time series (Pearson correlation of 0.98). Beyond that, Figure 4 clearly illustrates that the ratio of geosocial media posts related to COVID-19 decreased significantly over the course of the pandemic. Specifically, the percentage of relevant geosocial media posts gradually decreased from 5.7% at its peak in the first time frame, to 1.5% in the last time frame.

### Republican Counties

**Figure 5:**
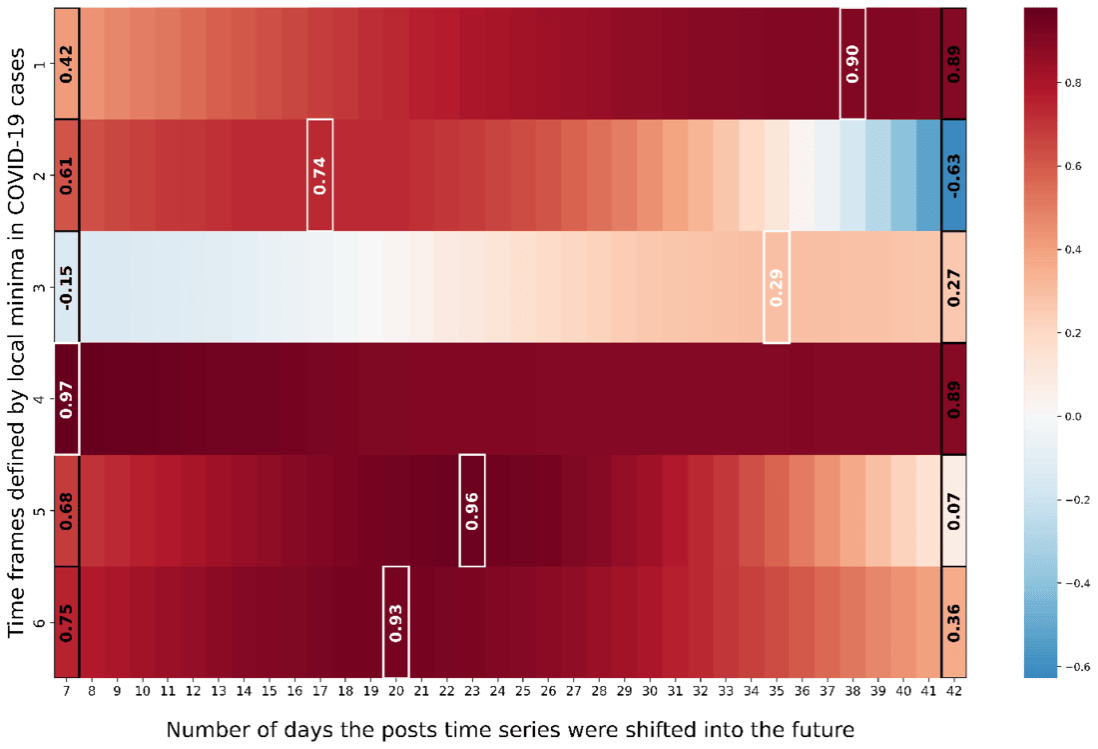
Depicting the Pearson correlation between COVID-19 cases and geosocial media post time series for each epidemiological wave when stepwise shifting the geosocial media post time series into the future for republican counties.

**Figure 6:**
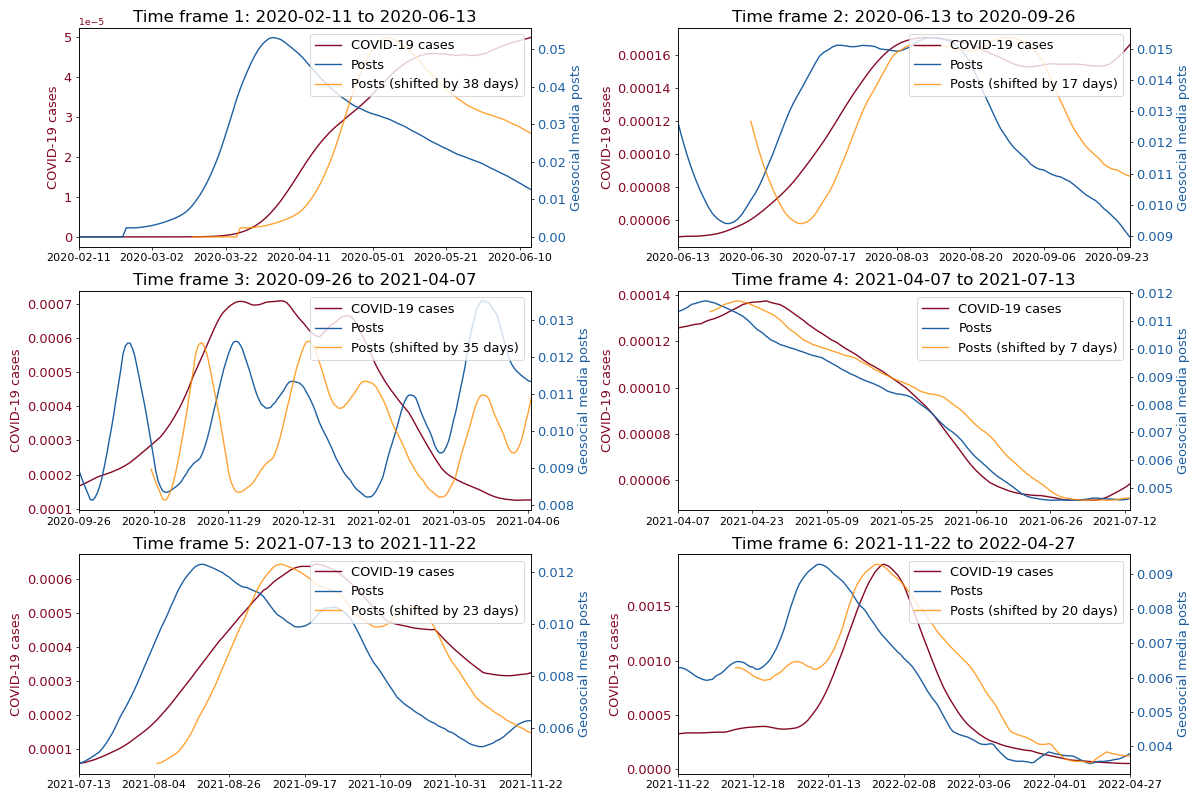
Depicting COVID-19 cases and the geosocial media posts time series and the shifted geosocial media posts time series across individual epidemic waves for republican counties.

Figure 5 illustrates for the republican counties that in 5 out of 6 time frames the post time series exhibited the highest Pearson correlation with the COVID-19 cases 7 to 38 days ahead of time (time frames 1, 2, and 4-6). Furthermore, for these time frames the Pearson correlations between posts shifted 7 to 38 days into the future and COVID-19 cases were between 0.74 and 0.97. Furthermore, Figure 6 showcases that for republican counties, early warning signals in geosocial media posts could be observed for time frames 1, 2 and 4-6. Similarly to the democrat county cluster, the COVID-19 cases wave in time frame 3 was not captured in advance by the geosocial media time series. The fact that all time frames besides time frame 3, lend themselves for early warning is also consistent with the results for the democrat counties. Furthermore, it appears that in the republican counties, geosocial media data preceded COVID-19 cases time series a few days more in advance. On average over all 5 time frames for which we attest early warning capabilities (time frames 1, 2, and 4-6), the mean temporal lag in democrat counties is 14.6 days (average correlation 0.94) and for 21 days republican counties (average correlation 0.9). Furthermore, it appears that the ratio of relevant posts decreased over time for republican counties from roughly about 5.3% to 0.9%.

### Swing Counties

**Figure 7:**
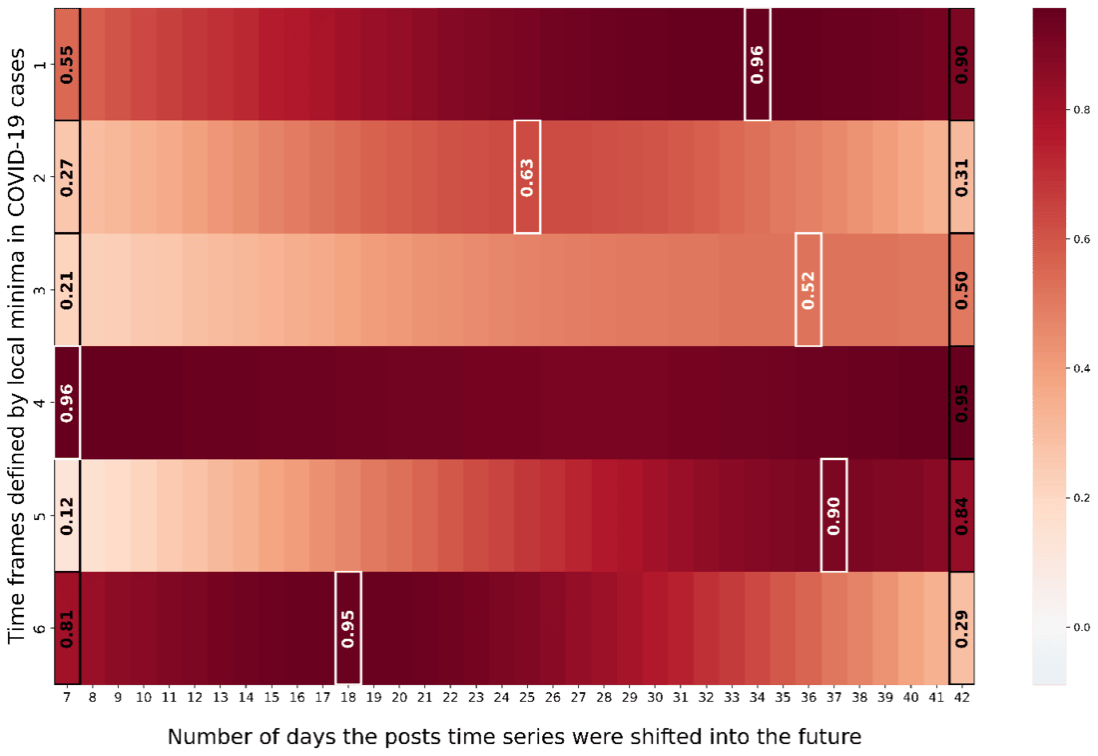
Depicting the Pearson correlation between COVID-19 cases and geosocial media post time series for each epidemiological wave when stepwise shifting the geosocial media post time series into the future for swing counties.

**Figure 8:**
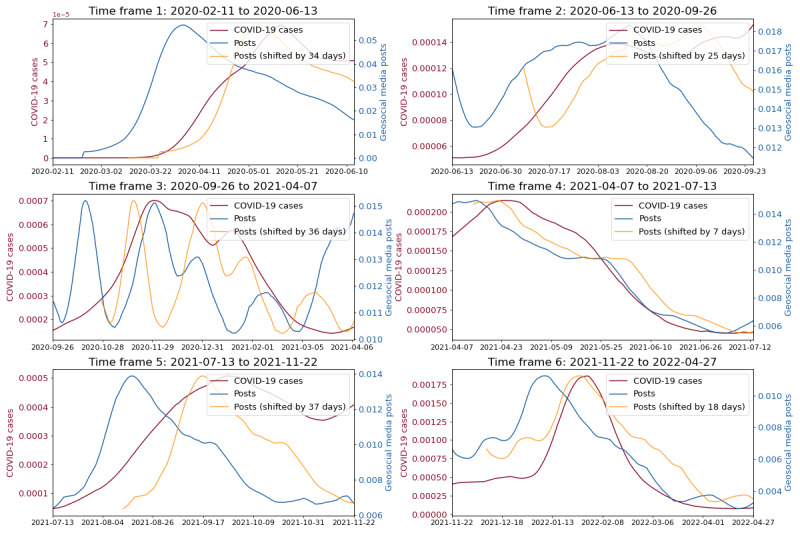
Depicting COVID-19 cases and the geosocial media posts time series and the shifted geosocial media posts time series across individual epidemic waves for swing counties.

Figure 7 illustrates for swing counties that shifting the posts time series between 7 and 37 days into the future achieved the highest correlation for all time frames. Furthermore, for all time frames the maximal Pearson correlations between geosocial media posts and COVID-19 cases ranged between 0.52 and 0.96. Beyond that, Figure 8 shows that the time frames 1, 2 and 4-6 exhibited clear early warning signals in geosocial media data ahead increases in COVID-19 cases. Similarly to the republican and democrat counties, the COVID-19 wave in time frame 3 was not clearly captured in advance by geosocial media data. However, similar, to republican counties, Figure 8 showcases for swing counties that there actually existed a signal in geosocial media data which is in line with the COVID-19 data in time frame 3. Nevertheless, the actual early warning capabilities are still limited due to noise in the signal which coincides with the COVID-19 infection of former President Donald Trump. Overall, the posts time series preceded COVID-19 cases in swing counties across all time frames, excluding the third, on average by 24.2 days. Also, the intensity with which geosocial media data appears to precede COVID-19 waves clearly decreased for swing counties over the course of the pandemic (from 5.6% to 1.1%).

## Discussion

### Principal Findings

The results of this study highlight how a deeper understanding of the relationship between COVID-19–related geosocial media data and confirmed COVID-19 cases, across politically distinct geographies, may help improve epidemiological early warning systems. Specifically, our analysis confirmed and expanded previous findings on the use of geosocial media posts as early indicators of disease activity [8-10,12]. However, we observed strong differences in the early warning capability of geosocial media data across different epidemiological waves. For example, geosocial media data were unable to reliably anticipate the third major COVID-19 wave, September 2020 to April 10, 2021 (time frame 3), across all political clusters. After significantly high COVID-19–related engagement on geosocial media in the first wave, it appears that the geosocial media signal lost some of its sensitivity in the third wave. The only event clearly detectable in COVID-19–related geosocial media posts in the third time frame is the COVID-19 infection of the former President Donald Trump in October 2020. The significance of this event might have reduced the sensitivity of the geosocial media users toward an increase in COVID-19 symptoms and infections. The reaction signal to this event was particularly visible in the republican and swing county clusters, while the democrat counties only registered a minor increase in geosocial media posts coinciding with the COVID-19 infection of President Trump. This further highlights how susceptible geosocial media data can be to politically charged trending topics and how these topics of interest might differ across political clusters. This is also in line with previous findings that the topics geosocial media users engage with and the language they use can differ depending on political beliefs [17-19]. Thus, we hypothesize that it might be key to identify different sets of keywords related to political beliefs and resulting trending topics, to capture geosocial media signals more accurately across political clusters. Therefore, future research should explore the influence of different geosocial media topics on early warning capabilities across political clusters and how such topics might change over time.

Furthermore, the findings of this study illustrate differences in the early warning capabilities of geosocial media posts for COVID-19 cases across counties with diverging political beliefs. This is particularly true for the number of days that geosocial media posts precede COVID-19 cases (temporal lag) and the Pearson correlation between these 2 time series for republican and democrat counties. For instance, geosocial media posts appear to anticipate COVID-19 cases in republican counties (21 days) on average 6.4 days earlier than in democrat counties (14.6 days). This difference in temporal lag might partly be caused by varying population densities between democrat and republican counties. In less densely populated republican counties [36], infection transmission might be slower [37], which could lead to a higher temporal lag between the onset of COVID-19 symptoms being observed and shared on geosocial media, to the eventual peak of infections in that region. However, it remains beyond the scope of this study to substantiate the actual underlying mechanisms which might cause these observed differences in early warning capability between political clusters. Despite that, the results of this study clearly emphasize the need to account for political beliefs in epidemiological early warning systems using geosocial media data. Nevertheless, the precise methodology to integrate political beliefs into real-time geosocial media-based early warning models remains the subject of future research.

The psychological effects of public health measures, such as lockdowns, might offer another explanation for the observed differences in early warning capabilities of geosocial media data across political clusters. These effects may be connected to the fact that public health measures were implemented and suspended at different points in time across political administrative areas. In this regard, Pettersen et al [38] associated more stringent public health and quarantine measures with increased mental distress in adults in Norway. Similarly, Ferwana and Varshney [39] observed a significant increase in visits to mental health facilities during the 2020 lockdown periods in the United States. While Ashokkumar and Pennebaker [14] even reported drops in analytical thinking and shifts in the emotional states of Reddit users coinciding with the start of lockdowns. Hence, it might be the case that the varying timing of public health measures across political regions caused various psychological effects, manifesting in changes of geosocial media behavior. However, our findings do not sufficiently verify this hypothesis. Although numerous studies have explored the psychological effects of public health measures, future research should focus on how these effects might influence the early warning capabilities of geosocial media data across the political spectrum.

In addition, we also found a clear decrease in the number of days with which geosocial media posts preceded COVID-19 cases and in the strength of the geosocial media post signal over time. Interestingly, yet to be explained, the decrease in temporal lag appears to be less pronounced in republican and swing counties. Nonetheless, this overall phenomenon might be caused by some sort of geosocial media and emotional COVID-19 fatigue. The association between self-reported depression symptoms and geosocial media usage [40], alongside potential factors contributing to social media fatigue [41-43] have already been explored in the context of the COVID-19 pandemic. For instance, recent findings by Li et al [43] indicate a direct relationship between social media overload during the COVID-19 pandemic and increased anxiety. Similarly, Sun and Lee [44] observe that COVID-19 information overload on social media directly contributes to fatigue toward pandemic related messages. Nevertheless, it remains beyond the scope of this study to substantiate whether the observed decreasing strength of the geosocial media post signal and temporal lag are robust and attributable to some form of geosocial media or COVID-19 fatigue. However, based on our observations, we advise caution, as the epidemiological early warning capabilities of geosocial media appear to change over time and depending on prevailing political beliefs. In this regard, it remains the task of future research to develop geosocial media-based early warning approaches, which can account for decreasing signal strength over time.

Furthermore, Howard et al [21] observed varying levels of misinformation and thus topics of interest, across states with different political beliefs. Interestingly, they found the highest rates of misinformation occurring in swing states. This is particularly noteworthy, as we found geosocial media data to be highly capable for early epidemiological warning in swing counties. Specifically, the average temporal lag of 24.2 days over all time frames in which we observed the highest early warning capabilities for swing counties, while mostly achieving high correlations (average correlation over all time frames with early warning capabilities 0.88). Thus, concluding from Howard et al [21] and our findings, it appears that it might not be the quality or factual correctness of the shared information on geosocial media that warrants its value for early warning purposes. Nevertheless, future research needs to further validate these findings in the context of different countries and their political ramifications as they might influence the relevance of shared information quality and factual correctness for epidemiological early warning capability.

### Data and Methods

We acknowledge that using a simple linear correlation measure might not always reflect the actual similarity between time series accurately. However, in preliminary analysis we also used different nonlinear correlation measures, which yielded only neglectable differences in the actual results. In addition, we also tested more advanced time series matching algorithms such as dynamic time warping [45], the Fréchet distance [46], or mutual information [47]. Nevertheless, neither nonlinear correlation measures nor more advanced comparison algorithms outperformed conventional linear correlation measures for most of our analyses. We evaluated the performance of these different methods in their ability to match the peaks and onsets of geosocial media signals and COVID-19 cases. Nonetheless, we acknowledge that the alignment of peaks and onsets is not always feasible, as the time it takes from the onset to the peak may vary between geosocial media signals and COVID-19 cases. As a result, for some epidemic waves the determined temporal lag might not reflect the actual real-world early warning capabilities of geosocial media data. Despite that, our main objective in this study was not to assess the exact temporal lag and correlations, but rather to provide an algorithmic way to compare the early warning capabilities of geosocial media data across political clusters.

In addition, there is a need to discuss the definition of epidemiological waves based on COVID-19 cases of the entire United States as one might argue that this decision might potentially have caused the observed variations in the number of days and the correlation between the geosocial media and the COVID-19 cases time series. The reason for this is that the COVID-19 waves can have different starting points and intensities across states [48] and as our results show also across political clusters (Figures 4, 6, and 8). Therefore, it might appear reasonable to assume that variation in the starting points and intensities caused the underlying observed differences in temporal lag and correlation between geosocial media posts and COVID-19 cases across political clusters. However, upon testing this hypothesis by defining COVID-19 waves individually for each political cluster, the fundamental results of our study remained unchanged. Although minor discrepancies were present in the temporal lag (primarily ranging from 1-2 days) and the correlations between COVID-19 cases and geosocial media posts, their differences persisted across political clusters and time frames in a similar manner. For example, republican counties still exhibited on average a higher temporal lag than democratic counties and the decrease in geosocial media signals was also still prevalent across political clusters.

In addition, it is important to consider the choice of keywords used for our analysis, as they strongly influence the observed results. One might argue that some keywords relevant to the discourse related to the COVID-19 pandemic were left out. However, in this analysis we mainly focused on gathering less polarized keywords, topics, and hashtags. The reason for this is that certain words, topics and hashtags were predominantly used by 1 political faction [17,18], which might indeed introduce bias into the final comparison between early warning capabilities across political clusters from the start. Concretely, keywords used predominantly in republican counties and less in democrat counties might directly influence differences in early warning capability across political clusters. Therefore, we decided to use a condensed set of keywords, which was to our knowledge mostly not inherently politically charged or biased.

Furthermore, we acknowledge that some keywords which we used in the semantic filtering process of the geosocial media posts, might not be only COVID-19 specific. However, we argue that for most words there exists a baseline signal of how often these words are being used. Therefore, our underlying assumption is that a real-world epidemiological event causes a significant spike in the usage of relevant keywords. Indeed, our results confirmed this assumption. We observed a baseline fluctuation in geosocial media posts and significant spikes in filtered posts, which in most cases preceded COVID-19 cases.

We also tried to improve the semantic filtering by leveraging machine learning approaches such as BERTopic or Latent Dirichlet Allocation [49,50]. However, due to performance issues with our large dataset (600+ GB) and based on the insufficient results for subsample experiments, we decided to stick to traditional keyword filtering. Nevertheless, in future work large language models [51] might be a possibility to improve the process of identifying relevant geosocial media posts.

### Limitations

The main limitation of this study stems from its retrospective nature. Our findings, while insightful for the past pandemic, may not be directly transferable to future epidemiological events. This limitation is partly due to the unpredictable nature of political polarization. Specifically, it is inherently difficult to predict whether a topic will become politically charged and, as a result, be discussed differently on social media across geographies with diverging political beliefs. In addition, social media behavior itself is influenced by various dynamic factors, for instance platform algorithms [52] or changing governance structures, which affect public engagement [53], all of which may differ significantly across social media platforms, future epidemiological events, and national borders. Although our study revealed differences in the epidemiological early warning capabilities of geosocial media data across US county-level political clusters, these results should be treated with caution when considering future-use cases.

### Conclusion

Our results confirmed the findings of previous research [9,10,12], demonstrating that geosocial media data can improve epidemiological early warning for consecutive waves of COVID-19 cases. In addition, we expand the existing literature by showing that the early warning capabilities of geosocial media data vary across US county clusters with differing political beliefs. For instance, geosocial media posts in republican counties (21 days) tend to precede increases in COVID-19 cases on average about 6.4 days earlier than in democrat counties (14.6 days). We hypothesize that this discrepancy in temporal lag between the geosocial media signal and the COVID-19 cases may stem from differences in the adoption of public health measures or population density variations across regions. In addition, we observed that the early warning capabilities of geosocial media data can be mitigated due to its susceptibility to a shift in trending topics and a decrease in signal strength over time.

Based on our findings, we would recommend that policy makers and researchers enhance and further investigate real-time geosocial media monitoring capabilities to improve epidemiological early warning systems. In addition, our findings suggest that it could be particularly beneficial for such systems to account for political beliefs prevalent across finer spatial scales such as county-level, given their potential to impact the early warning capabilities of geosocial media signals. Furthermore, since our results clearly highlight the value of geosocial media data for epidemiological early warning, we strongly encourage social media companies to grant researchers access to their data. Furthermore, future research should examine the early warning capabilities of different geosocial media topics specific to regional political beliefs and assess the transferability of our findings to other countries with different political environments. Furthermore, investigating the role of political communication strategies and potential improvements to social media algorithms to mitigate political polarization could enhance our understanding of how geosocial media data can be leveraged for future epidemiological events.

## Abbreviations

API: application programming interface
WHO: World Health Organization
